# Case report: combined transcatheter paravalvular leak closure and valve-in-valve transcatheter aortic valve replacement for treatment of severe regurgitation complicating transcatheter aortic valve replacement

**DOI:** 10.1093/ehjcr/ytag467

**Published:** 2026-06-24

**Authors:** Ashley P Armstrong, Luca Halalau, Luai Madanat, Ivan Hanson

**Affiliations:** Department of Internal Medicine, Corewell Health William Beaumont University Hospital, 3601 W 13 Mile Rd, Royal Oak, MI 48073, USA; Department of Liberal Arts & Sciences, Wayne State University, 4841 24th Ave, Detroit, MI 48208, USA; Department of Cardiovascular Medicine, Corewell Health William Beaumont University Hospital, 3601 W 13 Mile Rd, Royal Oak, MI 48073, USA; Department of Cardiovascular Medicine, Corewell Health William Beaumont University Hospital, 3601 W 13 Mile Rd, Royal Oak, MI 48073, USA

**Keywords:** Transcatheter aortic valve replacement (TAVR), Paravalvular leak (PVL), Amplatzer occluder, Valve-in-valve TAVR, Case report

## Abstract

**Background:**

Concurrent transvalvular regurgitation (TVR) and paravalvular leak (PVL) complicating transcatheter aortic valve replacement (TAVR0 is rare, and optimal management is not well described.

**Case Summary:**

A 74-year-old man with LVEF of 40% and prior TAVR (34-mm Medtronic Evolut) presented 8 years post-implantation with New York Heart Association (NYHA) class IV acute decompensated heart failure. Transthoracic echocardiography (TTE) identified severe bioprosthetic aortic regurgitation; transoesophageal echocardiography (TEE) delineated concomitant severe TVR and PVL. Computed tomographic angiography (CTA) identified a discrete paravalvular tunnel between two calcific annular nodules. Given the prohibitive surgical risk, he underwent single-session transcatheter PVL closure with an Amplatzer ductal occluder followed by valve-in-valve TAVR with an Edwards SAPIEN 3 Ultra RESILIA valve. AR pressure half-time improved from 217 to 436 ms, and invasive aortic diastolic pressure normalized from approximately 40–65 mm Hg. At 30-day follow-up, symptoms had improved to NYHA class II, with trace residual PVL and no TVR on TTE.

**Discussion:**

To the best of our knowledge, this represents one of the first reported cases of a combined single-session percutaneous approach, consisting of transcatheter PVL closure and valve-in-valve TAVR, to address mixed-mechanism bioprosthetic aortic regurgitation. TEE and CTA are essential for mechanism delineation. A combined transcatheter approach is feasible and effective in high-risk patients.

Learning Points

- Concomitant PVL and TVR should be considered in patients presenting with acute heart failure late after TAVR; TEE and CTA are essential for mechanism delineation and pre-procedural planning.
- With a paravalvular tunnel between bulky calcifications, a PVL-first strategy allows targeted defect closure and accurate reassessment of residual TVR before ViV TAVR.
- Single-session transcatheter PVL closure and ViV TAVR is feasible and effective in high-risk patients.

## Introduction

Late bioprosthetic valve dysfunction after transcatheter aortic valve replacement (TAVR) is increasingly encountered as procedural indications expand and long-term survival improves.^1^ Post-TAVR aortic regurgitation most commonly arises from paravalvular leak (PVL), caused by incomplete sealing between the prosthetic frame and the native annulus, and typically identified early after implantation. In contrast, transvalvular regurgitation (TVR) is less common and typically represents a late manifestation of intrinsic prosthetic leaflet failure.^2^

The concomitant occurrence of PVL and TVR is rare,^3^ and the optimal management strategy remains undefined. Current ESC/EACTS valvular heart disease guidelines recommend multidisciplinary Heart Team evaluation and consideration for redo intervention at experienced centres for symptomatic patients with severe bioprosthetic dysfunction.^4^

## Summary figure

**Figure:**
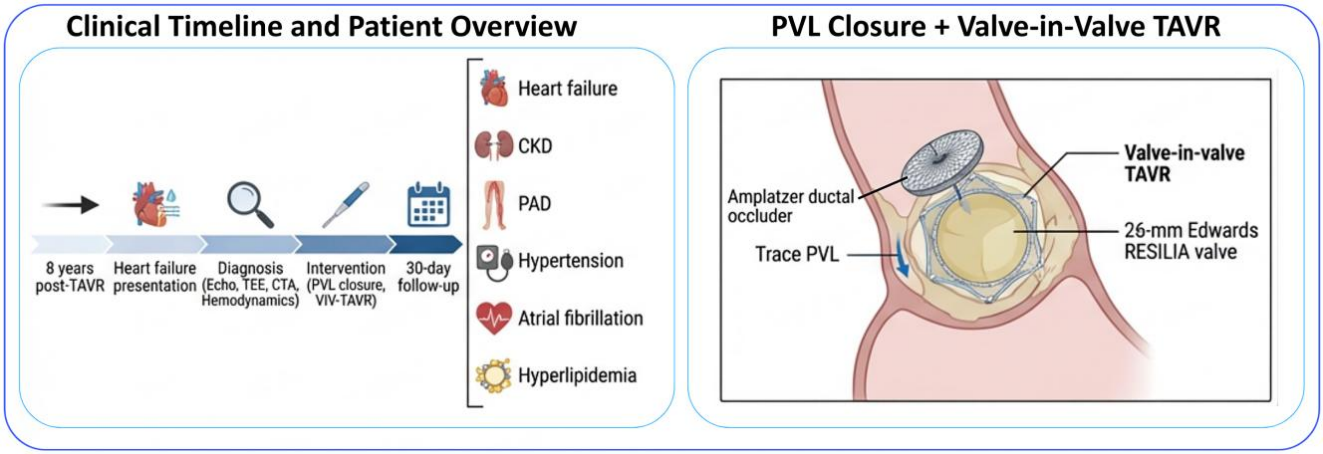


## Case presentation

A 74-year-old man with prior TAVR (34-mm Medtronic Evolut self-expandable valve), non-ischaemic cardiomyopathy (LVEF 40%), paroxysmal atrial fibrillation, CKD stage 3b, peripheral artery disease, hypertension, and hyperlipidaemia presented eight years after TAVR implantation with progressive dyspnoea, orthopnoea, and bilateral lower extremity oedema. Examination was notable for signs of volume overload and a new diastolic murmur. He was admitted with acute decompensated heart failure with New York Heart Association (NYHA) class IV symptoms. Notably, a transthoracic echocardiogram (TTE) two months prior had demonstrated normal bioprosthetic valve function.

**Figure 1:**
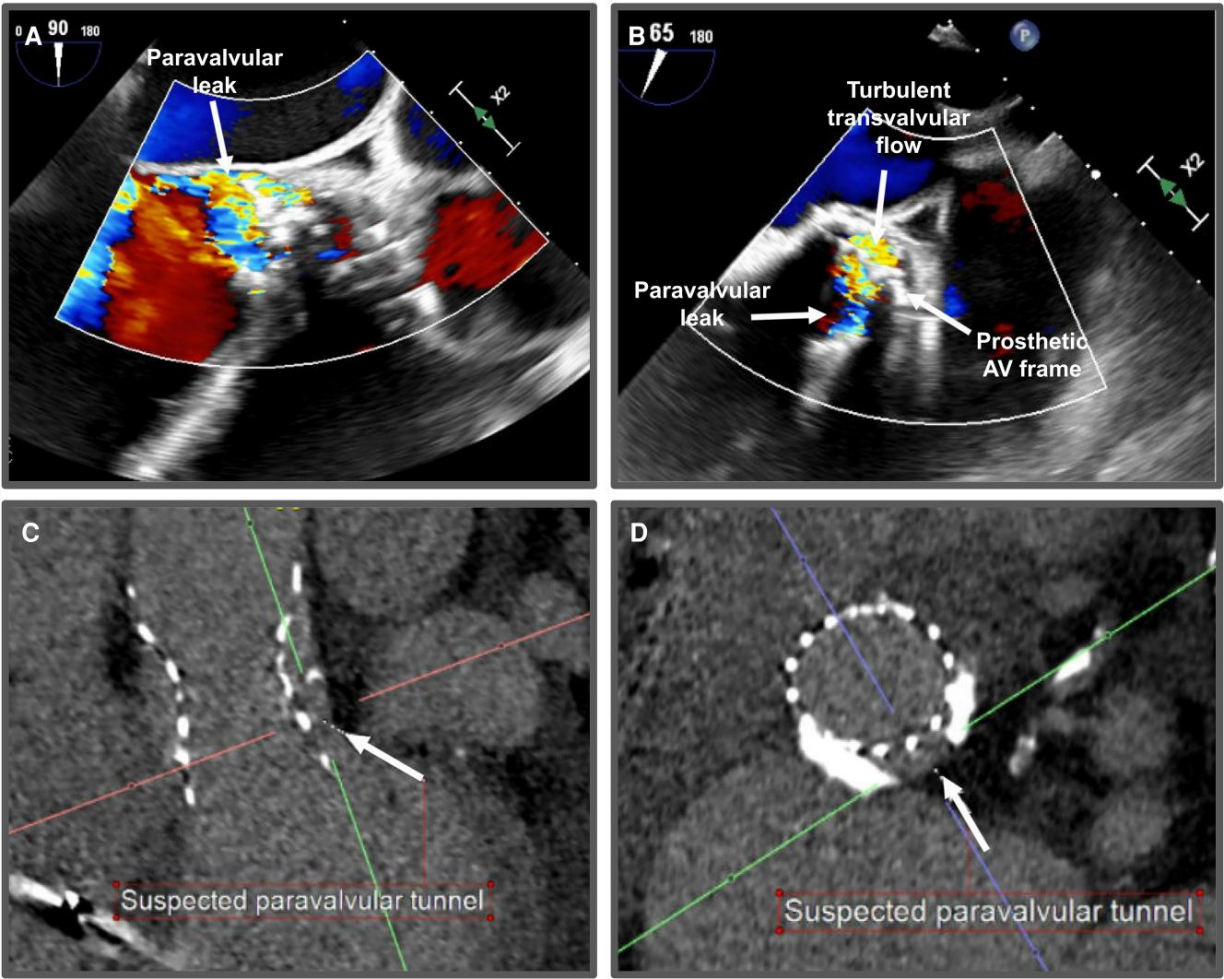
Multimodality imaging demonstrating concomitant paravalvular and transvalvular regurgitation: *(A and B)* TEE with colour Doppler, long- and short-axis views, demonstrating mixed-mechanism regurgitation, with severe paravalvular flow adjacent to the prosthetic frame, and severe transvalvular regurgitation centrally. *(C and D)* CTA, long- and short-axis reconstructions, demonstrating a self-expandable transcatheter aortic valve prosthesis with a discrete paravalvular regurgitant tunnel adjacent to the valve frame and located between two bulky calcific annular nodules.

TTE revealed severe bioprosthetic aortic regurgitation with a stable LVEF of 40%. Subsequent transoesophageal echocardiography (TEE) identified regurgitant jets both within and outside of the prosthetic frame, delineating concomitant severe PVL and TVR (*Figure 1A* and *B*, Supplementary material online, *Video S1*); however, the relative contributions of each component were incompletely characterized due to haemodynamic instability during the study. Invasive haemodynamic assessment confirmed markedly elevated filling pressures and post-capillary pulmonary hypertension (RA 10, PA 91/40, PCWP 24 mm Hg). Coronary angiography demonstrated non-obstructive disease. Computed tomographic angiography (CTA) demonstrated a discrete paravalvular tunnel along the left coronary cusp, between two bulky calcific annular nodules adjacent to the left main coronary artery (*Figure 1C* and *D*). A valve-to-coronary distance of greater than 4 mm was appreciated bilaterally, with the paravalvular tunnel located slightly posterior to the left main coronary artery rather than directly beneath it. CTA demonstrated no hypo-attenuated leaflet thickening or other features suggestive of leaflet thrombosis.

Following multidisciplinary Heart Team review, percutaneous intervention was recommended due to prohibitive surgical risk, with a PVL-first strategy selected.

**Figure 2:**
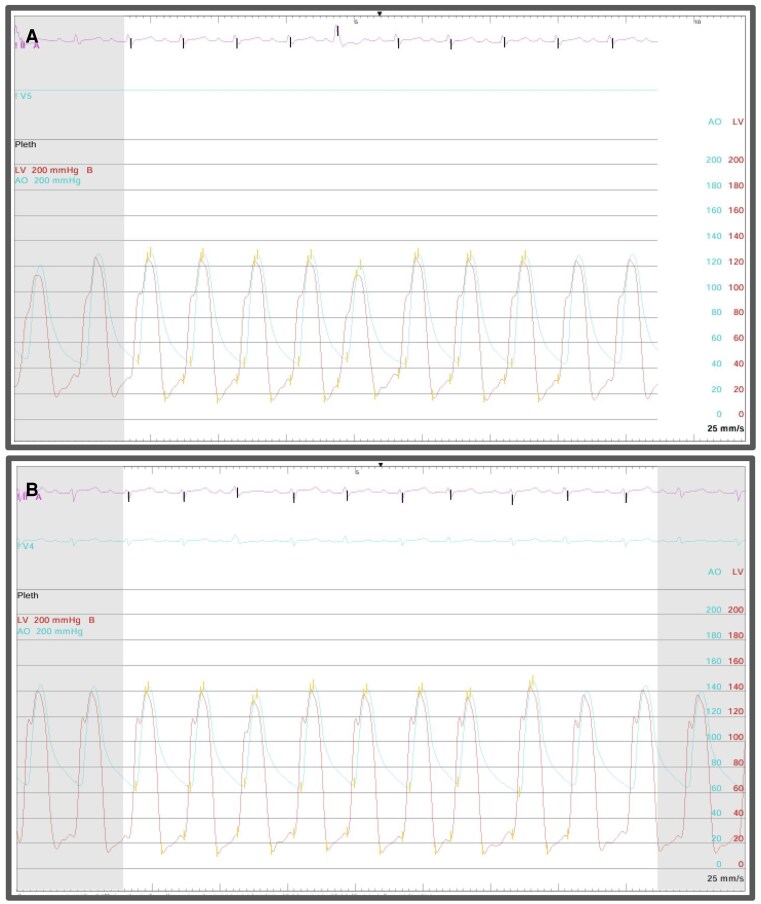
Invasive haemodynamic assessment of aortic (AO) and LV pressures. *(A*) Pre-procedural tracings demonstrating a wide AO pulse pressure with markedly reduced AO diastolic of approximately 40 mm Hg, reflecting significant diastolic runoff consistent with severe aortic regurgitation. *(B*) Post-procedural pressure tracings demonstrating normalization of AO diastolic pressure to approximately 60–70 mm Hg, confirming successful reduction of aortic regurgitation.

**Figure 3:**
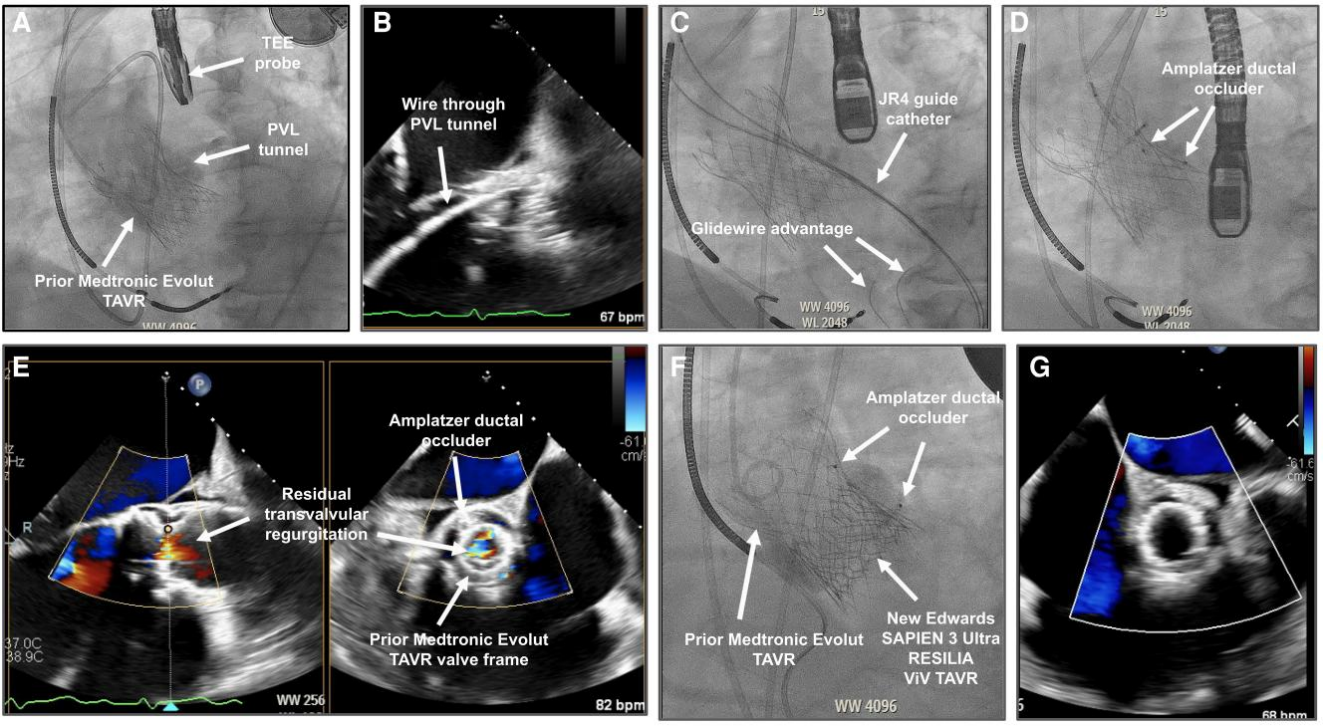
Procedural steps of percutaneous PVL closure and ViV TAVR. *(A*) Fluoroscopy during supravalvular aortography demonstrating the prior self-expandable Medtronic Evolut prosthesis, with localization of the PVL tunnel. *(B*) TEE demonstrating wire traversal through the PVL tunnel. *(C*) Fluoroscopy showing JR4 guide catheter and Glidewire Advantage positioned across the defect. *(D*) Fluoroscopy during deployment of a 6 × 4-mm Amplatzer ductal occluder across the PVL tunnel. *(E*) TEE with colour Doppler after PVL closure and prior to ViV TAVR demonstrating significant residual TVR. *(F*) Fluoroscopy following deployment of a 26-mm balloon-expandable Edwards SAPIEN 3 Ultra RESILIA valve within the prior Medtronic Evolut prosthesis. *(G*) Final TEE with colour Doppler demonstrating resolution of TVR with only mild residual PVL after the combined intervention.

The procedure was performed under general anaesthesia with TEE guidance, invasive haemodynamic monitoring, and cerebral embolic protection. Pre-procedural TEE confirmed severe PVL and TVR, with a mean transvalvular gradient of 10 mm Hg and AR pressure half-time of 217 ms. Invasive aortic and left ventricular pressure tracings demonstrated a wide aortic pulse pressure with a markedly reduced aortic diastolic pressure of approximately 40 mm Hg, reflecting significant diastolic runoff consistent with severe aortic regurgitation (*Figure 2A*). Supravalvular aortography localized the paravalvular defect along the left coronary cusp (*Figure 3A*). The defect was crossed with an AL1 catheter, and a Glide Advantage wire was advanced through the upper cells of the Evolut frame. Two 5-Fr JR4 catheters were then advanced across the defect, and a 6 mm × 4 mm Amplatzer ductal occluder—sized by CTA measurement of the tissue gap and TEE assessment of colour jet width—was deployed, with one disc positioned on the left ventricular side and the other on the aortic side, reducing PVL to trace (*Figure 3B–D*). Following PVL closure, TEE demonstrated a persistent central intra-prosthetic jet consistent with significant residual TVR, confirming the need to proceed with valve-in-valve TAVR (*Figure 3E*). A 26-mm Edwards SAPIEN 3 Ultra RESILIA balloon-expandable valve was advanced and successfully deployed (*Figure 3F*). Final aortography and TEE demonstrated complete resolution of TVR with only mild residual PVL (*Figure 3G*), and no coronary compromise. Normalization of aortic diastolic pressure to approximately 60–70 mm Hg on final invasive haemodynamic assessment provided objective confirmation of successful regurgitation reduction (*Figure 2B*).

The patient was discharged home in stable condition on post-procedure day 3. At the 30-day follow-up, he reported marked symptomatic improvement corresponding to NYHA class II. TTE demonstrated a well-seated valve-in-valve prosthesis with only trace residual PVL, no TVR, a mean transvalvular gradient of 5 mm Hg, and an AR pressure half-time of 436 ms.

## Discussion

Mixed-mechanism aortic regurgitation after TAVR presents both diagnostic and therapeutic challenges, as one regurgitant mechanism may obscure the other on standard imaging, and incomplete treatment risks substantial residual haemodynamic burden. The assessment of mixed-mechanism bioprosthetic regurgitation carries inherent imaging limitations: prosthetic frame artefact limits leaflet visualization, coexisting jets make individual quantification of PVL and TVR unreliable, and significant paravalvular flow may exaggerate the apparent severity of the transvalvular component. In patients presenting with late heart failure after TAVR, a systematic imaging approach is therefore essential—TTE as the initial screening tool, followed by TEE to characterize the mechanism and relative contributions of PVL and TVR, and finally CTA for anatomic delineation of paravalvular defect morphology, coronary risk assessment, and pre-procedural planning.

Late TVR after TAVR may result from progressive leaflet calcification, structural leaflet disruption or avulsion, leaflet thrombosis, pannus ingrowth, or frame deformation.^2,5^ The precise aetiology in this case was not definitively established due to limited leaflet visualization on TEE; however, the valve’s rapid deterioration from normal function to severe regurgitation over a 2-month period is most consistent with an acute structural event such as leaflet avulsion rather than gradual degeneration. Leaflet thrombosis was considered, but effectively excluded, as CTA demonstrated no hypoattenuated leaflet thickening. Infective endocarditis, another possible mechanism, was excluded based on negative blood cultures and absence of vegetations on multimodality imaging.

A PVL-first strategy was selected for several reasons. The well-defined paravalvular tunnel between bulky calcific nodules was thought unlikely to be sealed by the radial force of a second transcatheter valve alone. Additionally, deployment of a valve-in-valve prosthesis first may have impeded subsequent wire crossing of the paravalvular defect. Finally, elimination of paravalvular flow allowed for more accurate reassessment of TVR severity before committing to valve-in-valve TAVR. Device selection was guided by defect morphology and institutional practice. At the treating institution, ductal occluder devices are preferred for smaller, more focal defects given their lower-profile delivery system and greater flexibility—ideal for this defect bounded by bulky calcific nodules. Performing both interventions in a single session minimized cumulative procedural risk, consistent with the ESC Heart Team principle of individualized risk-stratified decision-making.^4^

Concomitant PVL and TVR after TAVR is rare, reported to have occurred in only 3% of patients in one large series.^3^ No established guidelines address the optimal management of this complication. To the best of our knowledge, this represents one of the first reported cases of a combined single-session percutaneous approach, consisting of transcatheter PVL closure and valve-in-valve TAVR, to address mixed-mechanism bioprosthetic aortic regurgitation. This case highlights that in patients presenting with late heart failure after TAVR, systematic multimodality imaging to delineate the precise regurgitant mechanism is a prerequisite to tailored intervention. When both PVL and TVR coexist, a transcatheter approach addressing both mechanisms in a single session is technically feasible and clinically effective.

As TAVR is increasingly performed in younger and lower-risk patients, the likelihood of requiring redo intervention during a patient’s lifetime grows correspondingly.^6^ When redo intervention is necessary, a single-session strategy that addresses both mechanisms of valve failure avoids the cumulative risk of staged procedures—an important consideration in the lifetime management of structural heart disease.

## Supplementary Material

ytag467_Supplementary_Data

## Data Availability

The data underlying this article are presented within the manuscript. The de-identified clinical data supporting the findings of this case report are available from the corresponding author upon reasonable request, subject to patient confidentiality and institutional approval.

## Lead author biography

Ashley P. Armstrong, DO, is an internal medicine resident at Corewell Health William Beaumont University Hospital in Royal Oak, Michigan, USA. She earned her Doctor of Osteopathic Medicine degree and a bachelor’s degree in Biochemistry and Molecular Biology from Michigan State University. Her clinical interests include cardiac imaging, novel transcatheter interventions, and cardiovascular prevention and screening. She plans to pursue fellowship training in cardiology and is committed to integrating evidence-based medicine into clinical practice.
